# MDH2 Promotes Hepatocellular Carcinoma Growth Through Ferroptosis Evasion via Stabilizing GPX4

**DOI:** 10.3390/ijms252111604

**Published:** 2024-10-29

**Authors:** Wenjia Yu, Yingping Li, Chengchang Gao, Donglin Li, Liangjie Chen, Bolei Dai, Haoying Yang, Linfen Han, Qinqin Deng, Xueli Bian

**Affiliations:** 1 The MOE Basic Research and Innovation Center for the Targeted Therapeutics of Solid Tumors, School of Basic Medical Sciences, Jiangxi Medical College, Nanchang University, Nanchang 330031, China; yuwenjia.email@foxmail.com (W.Y.); chengchanggao@163.com (C.G.); ldonglin0303@163.com (D.L.); chenliangjie9992@163.com (L.C.); daibolei@ncu.edu.cn (B.D.); yanghaoying2023@163.com (H.Y.); hanlinfen163@163.com (L.H.); dqqdengqinqin@163.com (Q.D.); 2 Shanxi Academy of Advanced Research and Innovation, Taiyuan 030032, China; liyingping1990@163.com

**Keywords:** tumor progression, ferroptosis, MDH2, HCC

## Abstract

The crosstalk between tumor progression and ferroptosis is largely unknown. Here, we identify malate dehydrogenase 2 (MDH2) as a key regulator of ferroptosis. MDH2 deficiency inhibits the growth of hepatocellular carcinoma (HCC) cells and enhances their sensitivity to ferroptosis induced by RAS-selective lethal 3 (RSL3), a compound known to cause ferroptosis. MDH2 knock-down enhances RSL3-induced intracellular reactive oxygen species, free iron ions and lipid per-oxides levels, leading to HCC ferroptotic cell death which is rescued by ferrostatin-1 and iron chelator deferiprone. Importantly, the inhibition of HCC cell growth caused by MDH2 deficiency is partially rescued by ferroptosis blockade. Mechanistically, MDH2 resists RSL3-induced ferroptosis sensitivity dependent on glutathione peroxidase 4 (GPX4), an enzyme responsible for scavenging lipid peroxides, which is stabilized by MDH2 in HCC. The protein expressions of MDH2 and GPX4 are positively correlated with each other in HCC cell lines. Furthermore, through our UALCAN website analysis, we found that MDH2 and GPX4 are highly expressed in HCC samples. These findings reveal a critical mechanism by which HCC evades ferroptosis via MDH2-mediated stabilization of GPX4 to promote tumor progression and underscore the potential of MDH2 inhibition in combi-nation with ferroptosis inducers for the treatment of HCC.

## 1. Introduction

Hepatocellular carcinoma (HCC) represents a significant global burden as one of the most prevalent malignant tumors, with increasing incidence and mortality rates, thereby presenting a major challenge to public health and clinical management [1,2,3]. Despite advancements in HCC treatment over the past few decades, the prognosis, particularly for patients in advanced stages, remains discouraging [4,5]. Therefore, a comprehensive understanding of HCC pathogenesis and the identification of novel therapeutic targets are of utmost importance.

In recent years, ferroptosis has attracted widespread attention as a new type of cell death. Compared with other common cell death modes (such as apoptosis, necrosis, and autophagy), ferroptosis is a cell death characterized by iron-dependent lipid peroxidation [6,7,8]. Excess iron leads to the generation of reactive oxygen species (ROS) through the Fenton reaction (the reaction of iron with hydrogen peroxide converts ferrous iron to ferric iron, while hydrogen peroxide generates hydroxyl radicals) [9,10]. Excessive ROS can induce lipid peroxidation on the cell membrane, thereby triggering ferroptosis [11]. Ferroportin (FPN) is responsible for exporting excess Fe^2+^ out of the cell, while auxiliary proteins such as ceruloplasmin and hephaestin assist in the oxidation of Fe^2+^ to Fe^3+^ for utilization by other tissues or cells [12,13,14]. Cells with elevated iron levels are more susceptible to ferroptosis, which can be blocked by iron chelators such as deferiprone (DFP) [15]. Cellular redox homeostasis is maintained by reduced glutathione (GSH) and glutathione peroxidase 4 (GPX4). GPX4 is the only enzyme that can utilize GSH to eliminate lipid peroxides [16,17]. Therefore, inhibition of lipoxygenase (such as non-selective inhibitor nordihydroguaiaretic acid and selective inhibitor zileuton) can effectively reduce the accumulation of lipid peroxides and thus inhibit ferroptosis [18]. In addition, depletion of GSH levels (such as erastin) [19] or the inhibition of GPX4 enzyme activity using compounds such as RAS-selective lethal 3 (RSL3), a known inducer of ferroptosis [20] can elevate intracellular lipid peroxide levels, ultimately triggering ferroptosis. For tumor cells, ferroptosis is of great significance because tumor cells rely on high levels of iron to promote invasive growth, making them more sensitive to ferroptosis [21]. Therefore, induction of ferroptosis has been considered a promising strategy against drug-resistant tumors, especially HCC [22]. However, further studies are needed to elucidate the regulatory mechanisms of ferroptosis in HCC.

Malate dehydrogenase 2 (MDH2) is a crucial enzyme in cellular metabolism as it serves as the terminal enzyme of the mitochondrial tricarboxylic acid (TCA) cycle. It can catalyze the conversion of L-malate to oxaloacetate, generating reducing equivalents [23]. Due to its significant role in the TCA cycle, MDH2 attracts widespread attention, and researchers found that it plays a pivotal role in tumor growth, metastasis, and chemotherapy resistance [24,25,26,27,28,29,30]. As a mitochondrial enzyme, MDH2 is involved in redox reactions and energy metabolism within mitochondria. Mitochondria play a crucial role in intracellular iron storage and metabolism [31,32,33]. Therefore, dysregulation of MDH2 may disrupt intracellular iron balance, potentially impacting the occurrence of ferroptosis. Recent investigations have suggested that reduced MDH2 expression in sarcopenia may promote muscle ferroptosis [34]. Metabolic enzymes can regulate various cellular activities depending on different stresses [35,36,37]. However, the specific role of MDH2 in HCC and its relationship with HCC ferroptosis remain largely unexplored.

In this study, we demonstrate that MDH2 promotes HCC ferroptosis evasion by inhibiting ubiquitination and degradation of GPX4. Furthermore, MDH2 deficiency sensitizes HCC cells to ferroptosis, which inhibits HCC tumor growth.

## 2. Results

### 2.1. MDH2 Is Highly Expressed in HCC and Promotes HCC Growth

**Figure 1:**
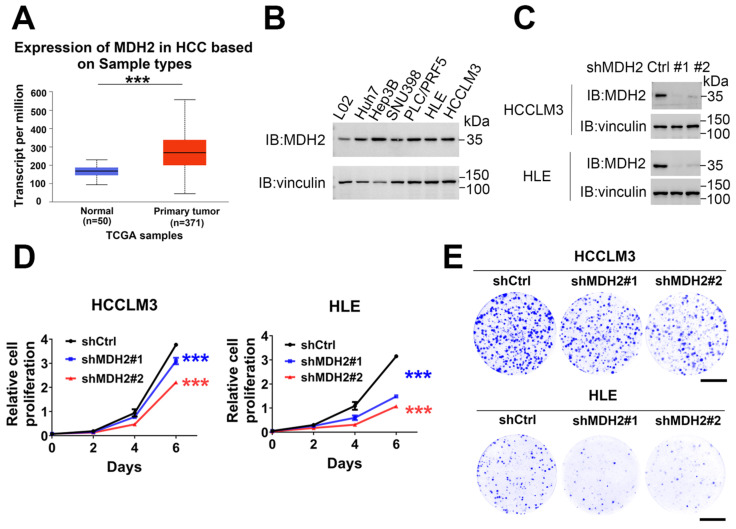
MDH2 promotes HCC cell proliferation. (**A**) Expression of MDH2 in normal liver tissues and primary HCC tissues obtained from UALCAN. (**B**,**C**) MDH2 protein levels were detected by WB, with vinculin as a loading control. (**D**) Cell proliferation assays were performed in control (shCtrl) and MDH2 knockdown (shMDH2#1 and shMDH2#2) HCC cells (3000 cells/well) for the indicated periods. Cells were stained with crystal violet, followed by dissolving with 10% glacial acetic acid, and absorbance at 595 nm was measured and calculated. (**E**) Indicated cells were seeded in the 6-well plate (500 cells/well), cultured for a few days (HCCLM3 cells for 10 days; HLE cells for 7 days), and then stained with crystal violet and photographed. Scale bar = 10 mm. (**D**) Data are represented as the mean ± standard deviation (SD) (*n* = 3); *** *p* < 0.0001 (two-way ANOVA). Abbreviations: Ctrl: control. HCC: hepatocellular carcinoma. MDH2: malate dehydrogenase 2. TCGA: The Cancer Genome Atlas Program.

To explore the function of MDH2 in HCC, firstly, we analyzed the expression of MDH2 using UALCAN (https://ualcan.path.uab.edu/analysis.html, accessed on 24 October 2024) and found that MDH2 was upregulated in HCC tissue samples compared with normal control tissues (Figure 1A). Furthermore, MDH2 protein levels were significantly higher in a panel of HCC cell lines (Huh7, Hep3B, SNU398, PLC/PRF5, HCCLM3 and HLE) than that in human normal hepatocytes (L02) (Figure 1B). These data suggest that MDH2 is upregulated in HCC. Subsequently, to determine the role of MDH2 on HCC cell growth, we stably knocked down MDH2 in HCCLM3 and HLE cells, and the knockdown efficiency was analyzed by Western blot (WB) (Figure 1C). Cell proliferation assays show that the knockdown of MDH2 significantly inhibited the growth of HCCLM3 and HLE cells (Figure 1D). To corroborate these results, we performed colony formation assays and obtained the same results (Figure 1E). Collectively, these results suggest that MDH2 is overexpressed in HCC and promoted HCC cell proliferation.

### 2.2. Knockdown of MDH2 Increases RSL3-Induced HCC Cell Death

**Figure 2:**
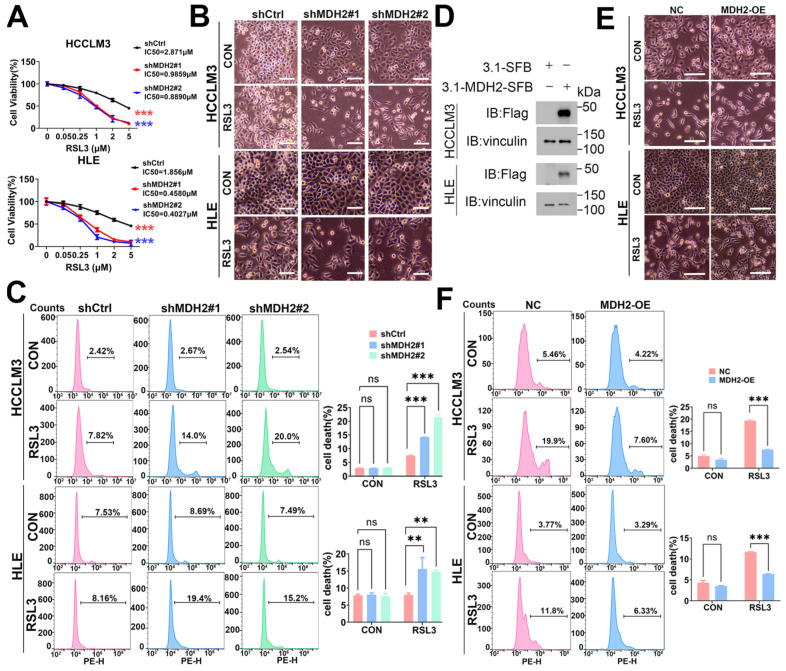
Knockdown of MDH2 increases RSL3-induced HCC cell death. (**A**) Control (shCtrl) and MDH2-knockdown (shMDH2#1 and shMDH2#2) HCC cells were treated with the indicated RSL3 concentrations for 48 h; and cell viability was assessed using the CCK-8 assay, and half-maximal inhibitory concentration (IC50) values were calculated. (**B**) Control (shCtrl) and MDH2-knockdown (shMDH2#1,shMDH2#2) HCC cells treated with or without 5 μM RSL3 for 24 h were photographed, and representative images are shown. Scale bar = 100 μm. (**C**) Control (shCtrl) and MDH2-knockdown (shMDH2#1,shMDH2#2) HCC cells treated with or without 5 μM RSL3 for 24 h were collected for PI staining and assayed by flow cytometry ((**C**), **Left**). Cell death rates were calculated and shown in a bar graph ((**C**), **Right**). (**D**) MDH2 protein levels in non-specific control (NC) and MDH2-overexpression (MDH2-OE) HCC cells were assessed by WB, with vinculin as a loading control. (**E**) Representative photographs of control (NC) and MDH2-overexpression (MDH2-OE) HCC cells treated with 5 μM RSL3 for 24 h. Scale bar = 100 μm. (**F**) Control (NC) and MDH2-overexpression (MDH2-OE) HCC cells were treated with 5 μM RSL3 for 24 h and then were collected for PI staining and assayed by flow cytometry ((**F**), **Left**). Cell death rates were calculated and are shown in bar graph ((**F**), **Right**). (**A**,**C**,**F**) Data are represented as the mean ± SD (*n* = 3); ns = no significance, ** *p* < 0.01, and *** *p* < 0.001 (two-way ANOVA for (**A**), one-way ANOVA for (**C**), and unpaired Student’s *t*-test for (**F**)). Abbreviations: CON: control. Ctrl: control. IC50: half-maximal inhibitory concentration. MDH2: malate dehydrogenase 2. NC: non-specific control. OE: overexpression. PE-H: phycoerythrin-height. RSL3: RAS-selective lethal 3.

Recent studies have revealed that the downregulation of MDH2 may contribute to the promotion of muscle ferroptosis in sarcopenia [34]. However, the relationship between MDH2 and ferroptosis in HCC remains to be explored. To investigate whether MDH2 plays a regulatory role in HCC ferroptosis, we treated HCC cells with different doses of the ferroptosis inducer RSL3, and the cell viability assays using cell counting kit-8 (CCK-8) reveal that the knockdown of MDH2 resisted RSL3-induced HCC cell viability (Figure 2A). Moreover, compared with wild-type (WT) HCC cells, MDH2-knockdown HCC cells had reduced cell adhesion, and more floating and dead cells were observed by microscopy after RSL3 treatment (Figure 2B). To further test whether MDH2 plays a pivotal role in RSL3-induced cell death, we next analyzed the proportion of cell death by propidium iodide (PI) staining, using flow cytometry. Figure 2C shows that the knockdown of MDH2 significantly promoted RSL3-induced HCCLM3 and HLE cell death. Additionally, to validate this finding, we stably overexpressed MDH2 in HCC cells (Figure 2D) and found that MDH2 overexpression impeded RSL3-induced HCC cell death (Figure 2E,F). Taken together, these findings demonstrate that MDH2 plays an inhibitory role in RSL3-induced cell death in HCC and that the knockdown of MDH2 increases the sensitivity of HCC to RSL3.

### 2.3. MDH2 Knockdown Increases RSL3-Induced HCC Cell Death via Upregulating ROS

**Figure 3:**
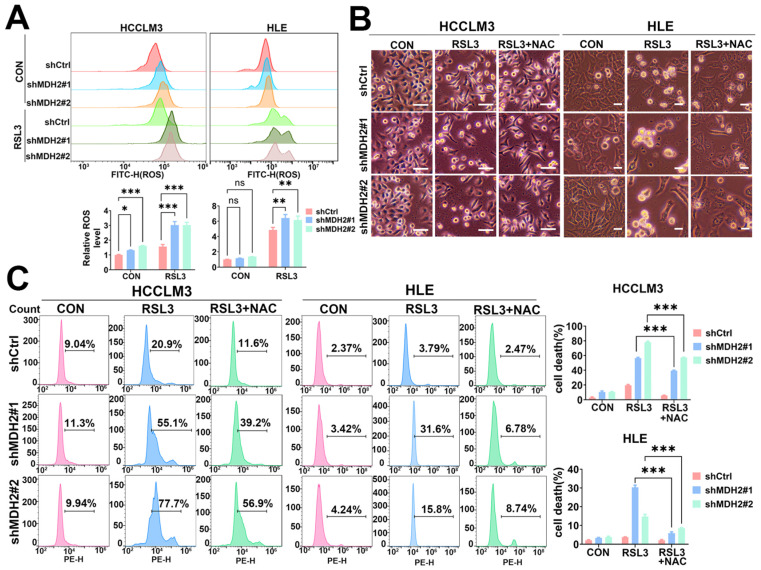
MDH2 knockdown sensitizes RSL3-induced HCC cell death via upregulated ROS. (**A**) Control (shCtrl) and MDH2-knockdown (shMDH2#1 and shMDH2#2) HCC cells treated with or without 5 μM RSL3 for 6 h were stained with 10 μM DCFH-DA probe for ROS assays using flow cytometry ((**A**), **Top**). Normalized ROS levels for the indicated cells are shown in the bar graph ((**A**), **Bottom**). (**B**) Control (shCtrl) and MDH2-knockdown (shMDH2#1 and shMDH2#2) HCC cells treated with or without 5 μM RSL3 or 5 mM NAC for 48 h were photographed, and representative images are shown. Scale bar = 100 μm. (**C**) Control (shCtrl) and MDH2-knockdown (shMDH2#1 and shMDH2#2) HCC cells treated with or without 5 μM RSL3 or 5 mM NAC for 48 h were collected for PI staining and assayed by flow cytometry ((**C**), **Left**). Cell death rates were calculated and are shown in bar graph ((**C**), **Right**). (**A**,**C**) Data are represented as the mean ± SD (*n* = 3); ns = no significance, * *p* < 0.1, ** *p* < 0.01, and *** *p* < 0.001 (one-way ANOVA for (**A**) and unpaired Student’s *t*-test for (**C**)). Abbreviations: CON: control. Ctrl: control. FITC-H: fuorescein isothiocyanate–height. MDH2: malate dehydrogenase 2. NAC: N-acetyl-L-cysteine. PE-H: phycoerythrin-height. ROS: reactive oxygen species. RSL3: RAS-selective lethal 3.

ROS-mediated lipid peroxidation is the central process underlying ferroptosis. During lipid peroxidation, ROS oxidize phospholipids, enzymes, and membrane receptors, as well as macromolecules like polyunsaturated fatty acid side chains and nucleic acids within the biological membranes, leading to the formation of lipid peroxides. These alterations disrupt the fluidity and permeability of the cell membrane, ultimately culminating in cell death [38]. Thus, assessing intracellular ROS content serves as an indicator of the extent of ferroptotic cell death. Next, we aimed to elucidate whether the knockdown of MDH2 enhances RSL3-induced HCC cell death through the upregulation of ROS. We employed a dichlorodihydrofluorescein diacetate (DCFH-DA) probe to monitor intracellular ROS levels and found that, compared with WT HCC cells, MDH2 knockdown cells significantly increased intracellular ROS levels upon RSL3 treatment (Figure 3A). Furthermore, we wondered whether the upregulated intracellular ROS levels in MDH2-knockdown cells induced by RSL3 are the key to HCC cell fate determination. We treated HCC cells with or without the ROS scavenger N-acetyl-L-cysteine (NAC), and the results showed that NAC significantly impeded RSL3-induced cell death in MDH2 knockdown HCC cells (Figure 3B,C). These results suggest that the knockdown of MDH2 enhances the susceptibility of HCC cells to RSL3-induced cell death through the upregulation of ROS, underscoring the critical role of MDH2 in preserving redox homeostasis and safeguarding HCC cells against oxidative stress and the cytotoxic effects induced by RSL3.

### 2.4. MDH2 Knockdown Sensitizes HCC to RSL3-Induced Ferroptosis

**Figure 4:**
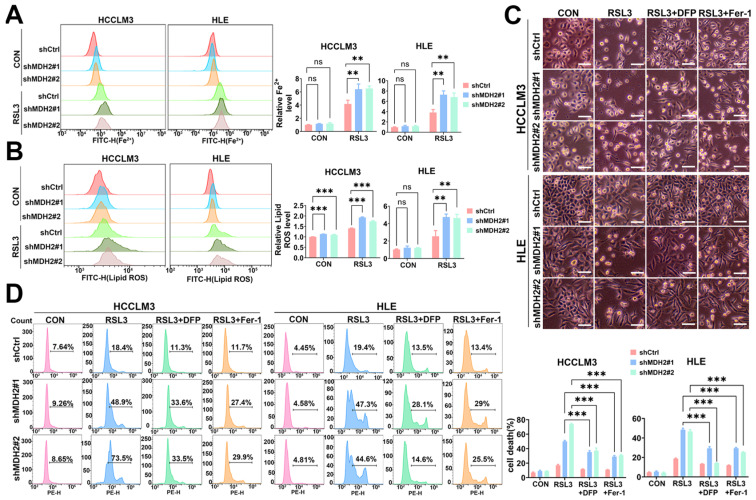
MDH2 knockdown sensitizes RSL3-induced ferroptosis. (**A**,**B**) Control (shCtrl) and MDH2-knockdown (shMDH2#1 and shMDH2#2) HCC cells treated with or without 5 μM RSL3 for 6 h were stained with 10 μM PGSK probe for Fe^2+^ assays (**A**) and stained with 10 μM C11-BODIPY^581/591^ for lipid ROS assays (**B**), followed by flow cytometry analysis ((**A**,**B**) **Left**). Normalized Fe^2+^ levels (**A**) and lipid ROS levels (**B**) for the indicated cells are shown in the bar graph ((**A**,**B**) **Right**). (**C**) Control (shCtrl) and MDH2-knockdown (shMDH2#1 and shMDH2#2) HCC cells treated with or without 5 μM RSL3, 2 μM Fer-1, or 50 μM DFP for 48 h were photographed, and representative pictures are shown. Scale bar = 100 μm. (**D**) Control (shCtrl) and MDH2-knockdown (shMDH2#1 and shMDH2#2) HCC cells treated with or without 5 μM RSL3, 2 μM Fer-1, or 50 μM DFP for 48 h were collected for PI staining and assayed by flow cytometry ((**D**), **Left**). Cell death rates were calculated and are shown in bar graph ((**D**), **Right**). (**A**,**B**,**D**) Data are represented as the mean ± SD (*n* = 3); ns = no significance, ** *p* < 0.01, and *** *p* < 0.001 (one-way ANOVA for (**A**,**B**,**D**)). Abbreviations: CON: control. Ctrl: control. DFP: deferiprone. Fer-1: ferrostatin-1. FITC-H: fuorescein isothiocyanate–height. MDH2: malate dehydrogenase 2. PE-H: phycoerythrin-height. RSL3: RAS-selective lethal 3.

The abovementioned experimental results showed that the downregulation of MDH2 enhances the sensitivity of HCC to RSL3-induced cell death by elevating ROS levels. Nevertheless, it is important to note that not all sources of ROS generation contribute equally to the demise of ferroptotic cells, despite ferroptosis being initiated by oxidative damage. Ferroptosis entails distinct molecular mechanisms to initiate and regulate the cell-death process, with iron-dependent ROS production and subsequent lipid peroxidation serving as the primary drivers [6,39]. Therefore, the aforementioned findings raise the question of whether MDH2 directly functions in the ferroptosis pathway. To address this question, we evaluated two direct indicators of ferroptosis: iron ions and lipid peroxides. Firstly, we employed the Fe^2+^ probe Phen Green SK (PGSK) and the lipid ROS probe C11-BODIPY^581/591^ to detect intracellular Fe^2+^ and lipid ROS contents by flow cytometry; the results show that the knockdown of MDH2 significantly increased intracellular free Fe^2+^ and lipid ROS levels under RSL3 treatment (Figure 4A,B). Furthermore, the ferroptosis inhibitor ferrostatin-1 (Fer-1), a lipophilic radical-trapping antioxidant, and the iron chelator DFP could rescue MDH2-knockdown HCC cell death induced by RSL3 (Figure 4C,D). These results reveal that MDH2 plays an important role in intracellular Fe^2+^ and lipid ROS homeostasis and further support the notion that MDH2 enhanced RSL3-induced cell death in HCC through the ferroptosis pathway.

### 2.5. Knockdown of MDH2 Inhibits HCC Cell Growth Partially Dependent on Ferroptosis

**Figure 5:**
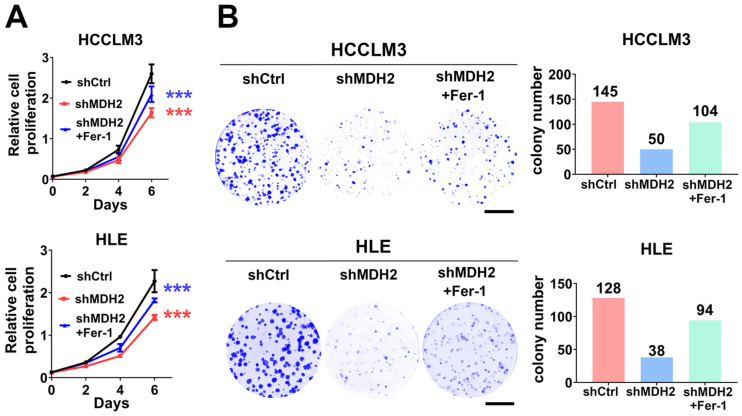
Knockdown of MDH2 inhibits HCC cell growth partially dependent on ferroptosis induction. (**A**) Cell proliferation assays were performed on control (shCtrl) and MDH2 knockdown (shMDH2#1 and shMDH2#2) HCC cells (3000 cells/well) with or without 0.5 μM Fer-1 for the indicated periods. Cells were stained with crystal violet, followed by dissolving with 10% glacial acetic acid, and absorbance at 595 nm was measured and calculated. (**B**) Control (shCtrl) and MDH2 knockdown (shMDH2#1 and shMDH2#2) HCC cells were seeded in the 6-well plate (500 cells/well) and cultured with or without 0.5 μM Fer-1 treatment for 10 days and then stained with crystal violet and photographed (**left**); the colony number was calculated and is shown (**right**). Scale bar = 10 mm. (**A**) Data are represented as the mean ± SD (*n* = 3); *** *p* < 0.001 (two-way ANOVA). Abbreviations: Ctrl: control. Fer-1: ferrostatin-1. MDH2: malate dehydrogenase 2.

Given the fact that our results showed that MDH2 is overexpressed in HCC and promotes HCC tumor growth and ferroptosis evasion. Therefore, we next wanted to know whether MDH2’s role in HCC cell growth is associated with ferroptosis. To solve this problem, we performed cell proliferation assays and found that ferroptosis inhibitor Fer-1 could partially rescue cell growth inhibition caused by MDH2 knockdown in HCCLM3 and HLE cells (Figure 5A). Furthermore, MDH2 knockdown significantly inhibited the colony-forming ability of HCCLM3 and HLE cells, and this inhibition was also partially abolished by Fer-1 treatment (Figure 5B). These results suggest that MDH2 knockdown inhibits HCC cell growth, which is partially dependent on ferroptosis pathway, and upregulation of MDH2 expression in HCC may promote ferroptosis evasion, thereby accelerating HCC tumor progression.

### 2.6. MDH2 Knockdown Sensitizes HCC to Ferroptotic Cell Death via GPX4

Ferroptosis, a form of regulated cell death, can be triggered through both exogenous and endogenous pathways. The exogenous pathway of ferroptosis is initiated by inhibiting cell membrane transporters such as the cystine/glutamate antiporter (System Xc-), or activating iron transporters such as serum transferrin and lactotransferrin. System Xc- is located on the cell membrane surface, with solute carrier family 7 member 11 (SLC7A11) being its primary functional subunit. SLC7A11 facilitates the transport of extracellular cystine into cells, which is crucial for the synthesis of GSH [40]. This process helps protect cells from damage caused by oxidative stress [41,42]. On the other hand, the endogenous pathway primarily triggers ferroptosis by inhibiting intracellular antioxidant enzymes such as GPX4 [43]. GPX4 is a core enzyme involved in regulating lipid peroxidation and is located in the mitochondria, cytoplasm, and nucleus. It utilizes electrons provided by GSH to convert phospholipid hydroperoxides into non-toxic phospholipid alcohols, thus neutralizing toxic peroxides and combating lipid peroxidation [8,44].

**Figure 6:**
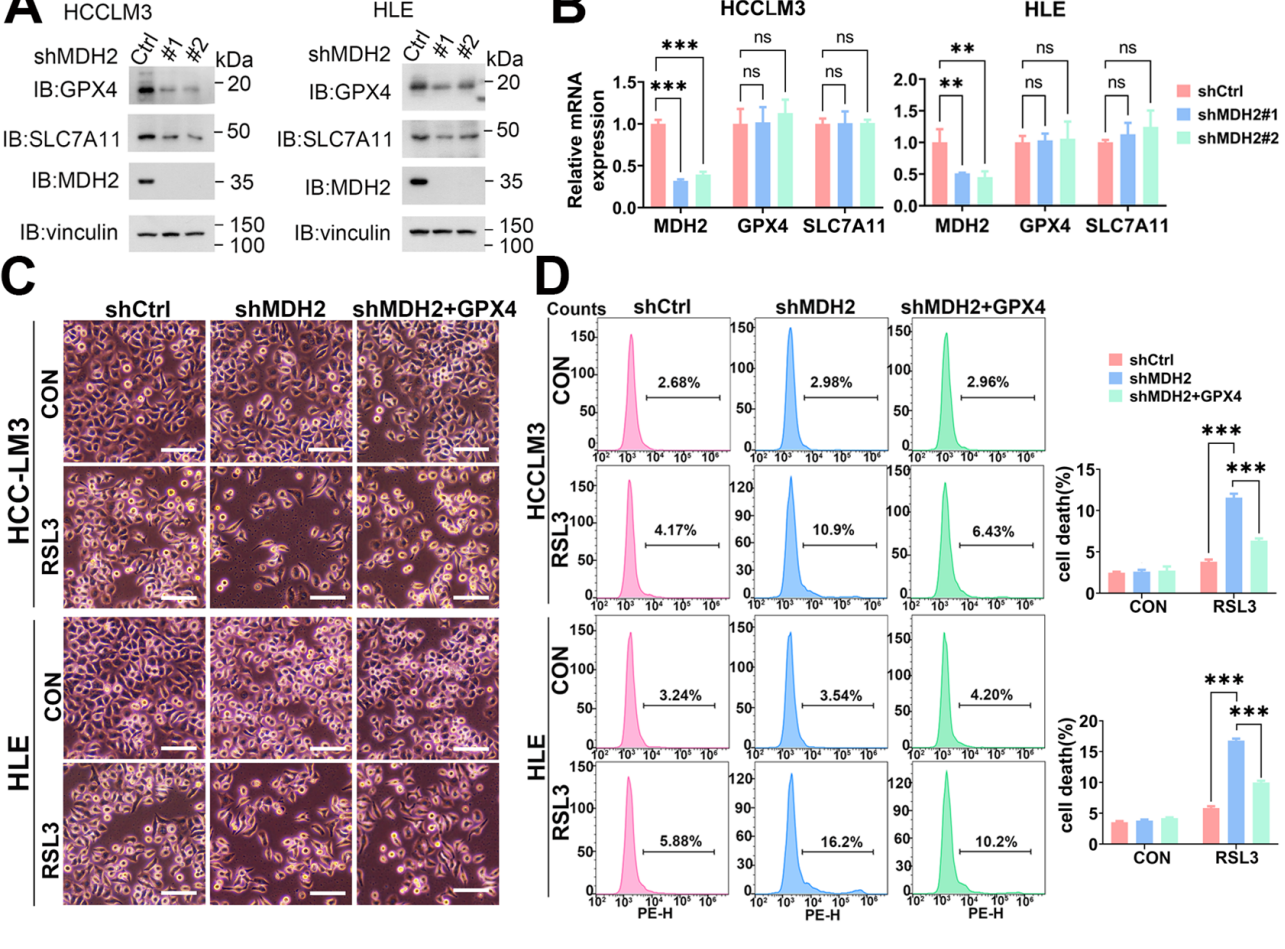
MDH2 induces ferroptosis via GPX4. (**A**) GPX4, SLC7A11, MDH2, and vinculin protein levels in control (Ctrl) and MDH2-knockdown (#1 and #2) HCC cells were assessed by WB. (**B**) *MDH2*, *GPX4*, and *SLC7A11* mRNA levels in control (shCtrl) and MDH2-knockdown (shMDH2#1 and shMDH2#2) HCC cells were determined by RT-qPCR. (**C**) Control (shCtrl) and MDH2 knockdown (shMDH2) HCC cells with or without GPX4 overexpression were treated with or without 5 μM RSL3 for 24 h and photographed; the representative images are shown. Scale bar = 100 μm. (**D**) Control (shCtrl) and MDH2 knockdown (shMDH2) HCC cells with or without GPX4 overexpression treated with or without 5 μM RSL3 for 24 h were collected for PI staining and assayed by flow cytometry ((**D**), **Left**). Cell death rates were calculated and are shown in bar graph ((**D**), **Right**). (**B**,**D**) Data are represented as the mean ± SD (*n* = 3); ns = no significance, ** *p* < 0.01, and *** *p* < 0.001 (one-way ANOVA for (**B**,**D**)). Abbreviations: CON: control. Ctrl: control. GPX4: glutathione peroxidase 4. MDH2: malate dehydrogenase 2. PE-H: phycoerythrin-height. SLC7A11: solute carrier family 7 member 11. RSL3: RAS-selective lethal 3.

To investigate the detailed mechanism of MDH2 in regulating ferroptosis in HCC cells, we analyzed the key proteins GPX4 and SLC7A11 in the ferroptosis pathway. The WB analysis showed that the knockdown of MDH2 in HCCLM3 and HLE cells significantly reduced the protein level of GPX4 and had little effect on SLC7A11 protein expression (Figure 6A). In fact, apoptosis pathways are often inactivated in cells resistant to targeted or chemotherapeutic drugs, and activation of other cell death pathways becomes the key to reversing drug resistance. Since tumor cells consume more iron than normal cells, resistant tumor cells are often particularly sensitive to ferroptosis [45,46,47]. Tumor cell resistance is believed to be dependent on GPX4, so inhibiting GPX4 activity can induce ferroptosis and reverse tumor cell resistance [48]. In order to elucidate how MDH2 regulates GPX4, we used quantitative reverse transcription polymerase chain reaction (RT-qPCR) assays, and the results show that MDH2 knockdown had no effect on *GPX4* and *SLC7A11* mRNA levels (Figure 6B), implying that MDH2 regulates GPX4 expression at the post-transcription level.

MDH2 plays a crucial role in catalyzing the dehydrogenation of malate, leading to the production of oxaloacetate and nicotinamide adenine dinucleotide, which subsequently generates nicotinamide adenine dinucleotide phosphate (NADPH) [49]. NADPH serves as an essential cofactor in the reduction process of GPX4, which facilitates the removal of peroxidized lipids from cells [50]. Notably, both MDH2 and GPX4 are localized in mitochondria, leading us to speculate that there may be a potential relationship between them that could impact ferroptosis in HCC. To verify whether MDH2 affects HCC cell ferroptosis via GPX4, we overexpressed GPX4 in MDH2-knockdown HCCLM3 and HLE cells, and our cell death analysis revealed that GPX4 overexpression significantly rescued MDH2-knockdown HCC cell death induced by RSL3 (Figure 6C,D). These results suggest that MDH2 knockdown sensitizes HCC cells to RSL3-induced ferroptotic cell death via downregulating GPX4 protein expression at the post-transcription level in HCC cells, supporting the view that MDH2 affects HCC cell ferroptosis via regulating GPX4.

### 2.7. MDH2 Interacts with and Stabilizes GPX4

**Figure 7:**
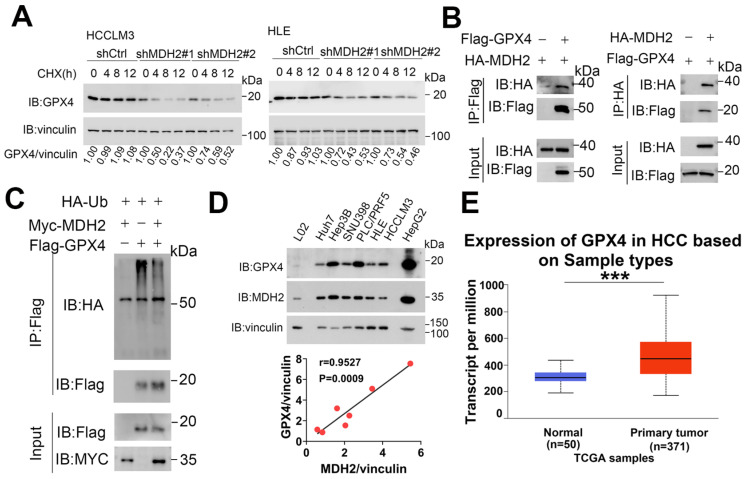
MDH2 interacts with and stabilizes GPX4. (**A**) Control (shCtrl) and MDH2 knockdown (shMDH2#1 and shMDH2#2) HCC cells treated with 100 μg/mL CHX for the indicated times were harvested and subjected to WB analysis with indicated antibodies. The quantification of normalized protein levels of GPX4/vinculin ratio are shown. (**B**,**C**) HEK-293T cells transiently transfected with indicated plasmids for 36 h were lysed and immunoprecipitated with the indicated antibodies, and the samples were subjected to WB analysis with indicated antibodies. (**D**) GPX4 and MDH2 protein levels were evaluated by WB, with vinculin as a loading control ((**D**), **Top**). Pearson correlation analysis of GPX4/vinculin and MDH2/vinculin protein expression in HCC cell lines is shown ((**D**), **Bottom**). (**E**) Expression of GPX4 in normal liver tissues and primary HCC tissues obtained from UALCAN. *** *p* < 0.001. Abbreviations: CHX: cycloheximide. Ctrl: control. GPX4: glutathione peroxidase 4. HCC: hepato-cellular carcinoma. IP: immunoprecipitation. MDH2: malate dehydrogenase 2. TCGA: The Cancer Genome Atlas Program. Ub: ubiquitin.

Protein expression can be regulated at post-transcriptional and post-translational levels. To examine the specific molecular mechanism of MDH2 in the regulating GPX4 protein level, we treated HCCLM3 and HLE cells with protein synthesis inhibitor cycloheximide (CHX) at different time points. Our WB analysis revealed that MDH2 knockdown significantly accelerated GPX4 degradation (Figure 7A), suggesting that MDH2 knockdown may decrease the expression level of GPX4 protein by inhibiting its stability. Co-immunoprecipitation (CO-IP) assays revealed the interaction between MDH2 and GPX4 (Figure 7B). Protein poly-ubiquitination is the signal to mediate protein degradation [51]; to corroborate these results, we immunoprecipitated exogenously overexpressed GPX4 and found that the overexpression of MDH2 inhibited the poly-ubiquitination level of GPX4 (Figure 7C), implying that MDH2 may enhance the stability of GPX4 by inhibiting its ubiquitination-mediated degradation. Furthermore, GPX4 protein levels were also higher in a panel of HCC cell lines (Huh7, Hep3B, SNU398, PLC/PRF5, HepG2, HCCLM3 and HLE) than in normal hepatocytes L02 cells (Figure 7C). It is noteworthy that the Pearson correlation analysis revealed a positive correlation between the expression levels of GPX4 and MDH2 in HCC cells (Figure 7D), indicating a potential functional relationship between these two proteins in HCC. To explore the functional significance of GPX4 in HCC, we analyzed the GPX4 expression in the UALCAN website and found that GPX4 expression was upregulated in tumor tissue samples from HCC patients compared with normal control tissue (Figure 7E).

In conclusion, this study elucidates the molecular mechanism of MDH2 in regulating GPX4 and its role in inhibiting RSL3-induced ferroptosis in HCC cells. These findings provide valuable insights into the regulatory mechanism of GPX4 and suggest that targeting the MDH2-GPX4 axis may be a promising strategy for HCC treatment.

## 3. Discussion

**Figure 8:**
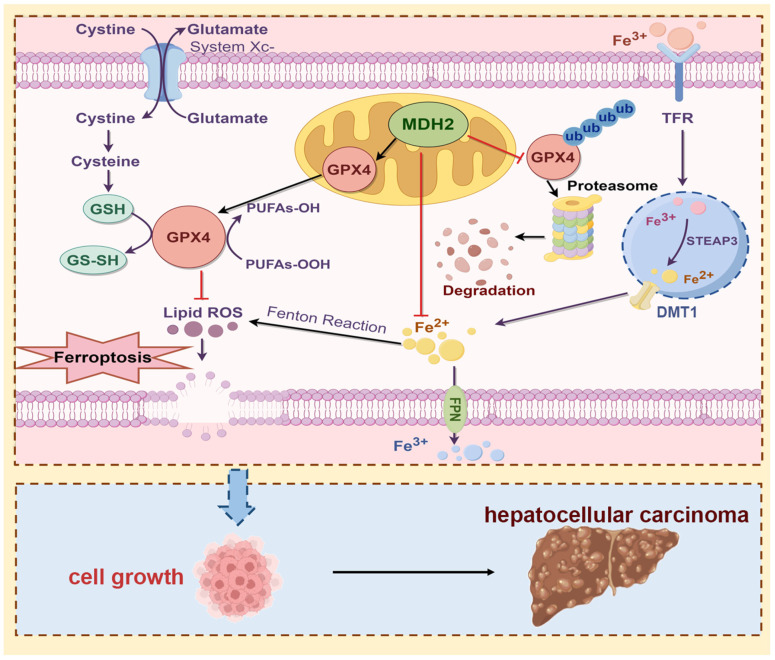
Schematic overview of the proposed mechanism whereby MDH2 promotes HCC progression via mediating ferroptosis evasion. Highly expressed MDH2 in HCC can reduce GPX4 ubiquitination and degradation to eliminate lipid ROS, leading to resistance to ferroptosis. On the other hand, MDH2 also plays a vital role in intracellular iron homeostasis to eradicate lipid ROS production via Fenton reaction inhibition. Overall, the function of ferroptosis evasion-mediated MDH2 promotes HCC progression. Abbreviations: DMT1: divalent metal transporter 1. FPN: ferroportin. GPX4: glutathione peroxidase 4. GSH: reduced glutathione. GS-SH: oxidized glutathione. MDH2: malate dehydrogenase 2. PUFAs-OH: polyunsaturated fatty acid alcohols. PUFAs-OOH: polyunsaturated fatty acid per-oxides. ROS: reactive oxygen species. STEAP3: six-transmembrane epithelial antigen of prostate 3. System Xc-: cystine/glutamate antiporter. TFR: transferrin receptor. ub: ubiquitin.

Ferroptosis is a type of programmed cell death that results from iron-mediated membrane lipid peroxidation [6,52]. Due to activated metabolic activity and higher ROS load in tumor cells, ferroptosis induction is a promising strategy for cancer treatment [39]. MDH2, the enzyme in TCA cycle, ranked first among ferroptosis-related differentially expressed genes in the GSE1428 dataset [34]. However, its role in tumor ferroptosis is elusive. In this study, through analyzing the TCGA database, we found that MDH2 and GPX4 were overexpressed in HCC tissues. The knockdown of MDH2 significantly inhibited HCC cell proliferation partially dependent on the ferroptosis pathway. The detailed mechanism reveals that the knockdown of MDH2 in HCC cells increased RSL3-induced intracellular ROS, Fe^2+^, and lipid ROS concentrations, thereby sensitizing HCC cells to RSL3-induced ferroptotic cell death. GPX4 and SLC7A11 are two key proteins that mediate ferroptosis evasion. The knockdown of MDH2 decreased the GPX4 protein level by impeding its protein stability, and MDH2 knockdown-mediated sensitization of HCC cells to RSL3-induced ferroptosis was rescued by overexpression of GPX4, suggesting that MDH2 regulates the HCC ferroptosis pathway mainly via the regulation of GPX4. Moreover, the protein levels of MDH2 and GPX4 were positively correlated in HCC cell lines. Taken together, our study reveals a new role of MDH2 in HCC ferroptosis and progression via regulating GPX4, and MDH2, which is highly expressed in HCC, may upregulate GPX4 to mediate ferroptosis evasion, leading to the failure of clinical cancer treatment. Therefore, MDH2 inhibition, in combination with ferroptosis inducers, may provide a promising strategy for HCC treatment (Figure 8).

Despite the fact that our results clearly show that MDH2 regulates HCC ferroptosis sensitivity via GPX4, there remain unanswered scientific questions and potential directions for further exploration. Ferroptosis is a form of programmed cell death characterized by abnormal increases in iron-dependent lipid peroxidation [6]. Within the acidic environment of endosomes, the six-transmembrane epithelial antigen of prostate 3 (STEAP3) reduces Fe^3+^ to Fe^2+^. Divalent metal transporter 1 (DMT1) facilitates the transport of Fe^2+^ into the cytoplasm, where it partakes in various physiological functions [53,54,55]. Excess iron leads to the generation of ROS through the Fenton reaction [39]. In this study, we observed that the knockdown of MDH2 in HCC cells resulted in the accumulation of intracellular Fe^2+^ levels in HCC cells upon RSL3 treatment. Due to the important role of MDH2 in cellular metabolism, MDH2 deficiency may lead to the dysregulation of cellular iron metabolism or iron transport, and further studies should clarify this detailed mechanism. Moreover, recent studies have demonstrated that modulating the malate–aspartate shuttle pathway through the inhibition of glutamate-oxaloacetate transaminase 2 or MDH2 can trigger cell death and diminish adenosine triphosphate levels in non-small cell lung cancer, akin to the impact of glutaminase 1 inhibition [56]. Furthermore, glutamine deprivation results in the decreased expression of GPX4, implying that targeting glutaminolysis influences the chemoresistance of cancer cells. The elevation of nuclear factor erythroid 2–related factor 2 (NRF2)–antioxidant pathway markers, heightened glutaminolysis, and elevated GPX4 levels collaboratively bolster chemoresistance in pancreatic cancer cells, shedding light on the potential contributions of glutamine metabolism and NRF2 signaling in the regulation of GPX4 function by MDH2 and chemoresistance in HCC [57].

Furthermore, mitochondria play an important role in ferroptosis, so it is crucial to conduct further investigations to elucidate whether the knockdown of the mitochondrial enzyme MDH2 influences the occurrence of ferroptosis by perturbing mitochondrial integrity and function. These studies will contribute to a more comprehensive understanding of the role of MDH2 in regulating ferroptosis and its potential impact on mitochondrial dynamics. In addition, although the findings suggest that MDH2 plays a role in ferroptosis resistance by stabilizing GPX4, the precise regulatory mechanism of MDH2 on GPX4 stability requires further study.

## 4. Materials and Methods

### 4.1. Cell Lines and Cell Culture

Hepatocellular carcinoma (HCC) cell lines (Huh7, Hep3B, SNU398, PLC/PRF5, HCCLM3, HLE, and HepG2), human normal hepatocytes (L02), and human embryonic kidney cells (HEK-293T) were purchased from the Cell Bank of the Type Culture Collection Committee of the Chinese Academy of Sciences. Cells were maintained in Dulbecco’s modified Eagle medium (DMEM, Solarbio, Beijing, China, Cat# 11965) supplemented with 10% fetal bovine serum (Excell Bio, Shanghai, China, Cat# FSP500) at 37 °C in a 5% CO_2_ atmosphere. The cells were replaced with culture medium every 1–2 days, and after growing to the logarithmic phase (cells grow to about 80%), follow-up experiments or subcultures could be performed.

### 4.2. Regents and Antibodies

Regents used in this study included the following: ABI QuantStudio 7 Flex with SYBR kit (TransGen, Beijing, China, Cat# AQ601-02); C11-BODIPY^581/591^ (MCE, Shanghai, China, Cat# HY-D1691); cell counting kit-8 (CCK-8, TargetMol, Boston, MA, USA, Cat# C0005); cycloheximide (CHX, MCE, Cat# HY-12320); dichlorodihydrofluorescein diacetate (DCFH-DA, MCE, Cat# HY-D0940); deferiprone (DFP, Abmole, Houston, TX, USA, Cat# M2617); dodecyl sulfate sodium (SDS, Solarbio, Cat# S8010); enhanced chemiluminescence (ECL) chemiluminescence solution kit (Meilunbio, Dalian, China, Cat# MA0186); ferrostatin-1 (Fer-1, Abmole, Cat# M2698); M5 HiPer One-step RT-PCR Kit (Mei5bio, Beijing, China, Cat# MF051-01); M5 Super plus qPCR RT kit with gDNA remover (Mei5bio, Cat# MF166-plus-T); N-Ethylmaleimide (NEM, Solarbio, Cat# N8760); N-acetyl-L-cysteine (NAC, MCE, Cat# HY-B0215); paraformaldehyde (Solarbio, Cat # P1110); phenylmethylsulfonyl fluoride (PMSF, Dingguo Biotechnology, Wuhan, China, Cat# 329-98-6); propidium iodide (PI, TargetMol, Cat# T2130); Phen Green SK (PGSK, MCE, Cat# HY-126823); polybrene (MCE, Cat# HY-112735); puromycin (Solarbio, Cat# P8230); RAS-selective lethal 3 (RSL3, CSNpharm, Arlington Heights, IL, USA, Cat# CSN17581); radio-immunoprecipitation assay (RIPA) cell lysis buffer (Solarbio, Cat# R0100); superfectin II DNA transfection reagent (Pufei Biotechnology, Shanghai, China, Cat# 2102-100); TRIzol reagent (Invitrogen, Carlsbad, CA, USA, Cat# 15596-026); and Z-Leu-Leu-Leu-CHO (MG132, Biovision, San Francisco, CA, USA, Cat# 1791-5). 

The antibodies were as follows: Flag (Sigma-Aldrich, Beijing, China, Cat# 66008-3Ig, used at 1:5000); glutathione peroxidase 4 (GPX4, Proteintech, Wuhan, China, Cat# 67763-1-Ig, used at 1:1000); HA (Santa Cruz, Dallas, TX, USA, Cat# sc-7392, used at 1:5000); horseradish peroxidase (HRP)-conjugated goat anti-mouse or rabbit antibody (Santa Cruz, used at 1:5000); malate dehydrogenase 2 (MDH2, Proteintech, Cat# 15462-1-AP, used at 1:4000); MYC (Proteintech, Cat# 60003-2-Ig, used at1:5000); solute carrier family 7 member 11 (SLC7A11, Proteintech, Cat# 18790-1-AP, used at 1:4000); and vinculin (Santa Cruz, Cat# sc-73614, used at 1:8000).

### 4.3. Western Blot (WB)

First, cells were lysed with RIPA cell lysis buffer (Solarbio, Cat# R0100) containing 1% PMSF (protease inhibitor) (Dingguo Biotechnology, Cat# 329-98-6) for 30 min at 4 °C, and then the cells were transferred into 1.5 mL Eppendorf (EP) tube to centrifuge at 12,500× *g* for 20 min at 4 °C. After centrifugation, the supernatant was transferred into a new EP tube. Subsequently, 2× loading buffer was added at a 1:1 ratio, mixed well, and boiled for 10 min. Samples were then subjected to sodium dodecyl-sulfate polyacrylamide gel electrophoresis (SDS-PAGE). After proteins were transferred to a polyvinylidene fluoride (PVDF) membrane (Millipore, Mannheim, Germany, Cat# 03010040001), the PVDF membrane was blocked in 5% skimmed milk powder (Solarbio, Cat# D8340) for 1 h at room temperature and then incubated with selected antibodies. After washing with tris-buffered saline-tween 20 buffer, ECL chemiluminescence solution kit (Meilunbio, Cat# MA0186) was used for color development, and finally imaging and storage were performed. The primary antibodies and concentrations used for WB were as follows: Flag (Sigma-Aldrich, Cat# 66008-3Ig, used at 1:5000); GPX4 (Proteintech, Cat# 67763-1-Ig, used at 1:1000); HA (Santa Cruz, Cat# sc-7392, used at 1:5000); HRP-conjugated goat anti-mouse or rabbit antibody (Santa Cruz, used at 1:5000); MDH2 (Proteintech, Cat# 15462-1-AP, used at 1:4000); MYC (Proteintech, Cat# 60003-2-Ig, used at1:5000); SLC7A11 (Proteintech, Cat# 18790-1-AP, used at 1:4000); and vinculin (Santa Cruz, Cat# sc-73614, used at 1:8000).

### 4.4. Construction of Stable MDH2 Knockdown Cell Line

The lentivirus was packaged in HEK-293T cells. Briefly, HEK-293T cells were seeded and transfected with the target plasmid (plko.1-puromycin or shMDH2 plasmid) and lentivirus packing plasmids PSPAX2 and PMD2.G. After transfection for 48 h, the supernatant was collected and filtered with a 0.45 μm filter to remove cell debris, and 5 μg/mL polybrene (MCE, Cat# HY-112735) was added for the infection of HCC cells. Forty-eight hours after infection, 2 μg/mL puromycin (Solarbio, Cat# P8230) was added to the culture medium of HCC cells for 2 days to kill the uninfected cells.

Primer sequence for MDH2 shRNA:

shMDH2#1:
CCGGTGGCCAGTTTCCTTAATTTATCTCGAGATAAATTAAGGAAACTGGCCATTTTTG (Former Primer 5′-3′),
AATTCAAAAATGGCCAGTTTCCTTAATTTATCTCGAGATAAATTAAGGAAACTGGCCA (Reverse Primer 5′-3′).
shMDH2#2:
CCGGTCATTGCCAATCCGGTTAATTCTCGAGAATTAACCGGATTGGCAATGATTTTTG (Former Primer 5′-3′),
AATTCAAAAATCATTGCCAATCCGGTTAATTCTCGAGAATTAACCGGATTGGCAATGA (Reverse Primer 5′-3′).

### 4.5. Transient Transfection of Plasmid

Cells were seeded and transfected when the cell density was approximately 80%. The superfectin II DNA transfection reagent (Pufei Biotechnology, Cat# 2102-100) was used for transient transfection of plasmid in HCC cells. Briefly, 125 μL of serum-deficient DMEM medium (Solarbio, Cat# 11965) was added to tubes A and B, respectively, and then 9 μL of superfectin II DNA transfection reagent (Pufei Biotechnology, Cat# 2102-100) was added to tube B. Tube A was provided with 3 μg of plasmids (3.1-SFB or 3.1-MDH2-SFB plasmids), and the liquid from tube B was carefully added to tube A before being gently mixed. After standing for 15 min, the transfection mixture was evenly dispersed into the cells, and cells were cultured 12–16 h. Then, the medium was replaced with fresh culture medium for another culture for 12–24 h.

### 4.6. Cell Proliferation Assay

HCCLM3 and HLE cells (3000 cells per well) were seeded into 24-well plates and cultured for 0, 2, 4, and 6 days. The cells were harvested and washed three times with phosphate-buffered saline (PBS), followed by fixation with 4% paraformaldehyde (Solarbio, Cat# P1110) for 30 min at room temperature. After fixation, cells were washed with PBS and stained with 500 μL crystal violet for 2 min. Then, the free crystal violet was removed, and 200 μL of 10% glacial acetic acid per well was added to the 24-well plate. After being thoroughly shaken to dissolve and elute for 1 h, the crystal violet dissolved in glacial acetic acid and was transferred to a 96-well plate. The optical density (OD) value, which is a logarithmic measurement of the percent transmission, was measured on a microplate reader at a wavelength of 595 nm, data were calculated, and a growth curve was plotted based on the OD values.

### 4.7. Colony Formation Assay

The cells were digested, and 500 cells per well were seeded into 6-well plates. The cells were then cultured at 37 °C and 5% CO_2_ for a few days. Subsequently, the cells were harvested and washed three times with PBS, followed by fixation with 4% paraformaldehyde (Solarbio, Cat# P1110) for 30 min at room temperature. Then, cells were stained with 0.1% crystal violet in 20% methanol for 15 min and washed with PBS to remove the free crystal violet and then photographed.

### 4.8. Quantitative Reverse Transcription Polymerase Chain Reaction (RT-qPCR)

Total RNA was extracted from cells using TRIzol reagent (Invitrogen, Cat# 15596-026), according to the manufacturer’s instructions. RNA integrity, quantity, and purity were checked using a NanoDrop 2000c spectrophotometer (Thermo Fisher Scientific, Waltham, MA, USA, Cat# ND-2000C). Genomic DNA was removed using M5 Super plus qPCR RT kit with gDNA remover (Mei5bio, Cat# MF166-plus-T). Total RNA was then reverse transcribed into cDNA using the M5 HiPer One-step RT-PCR Kit (Mei5bio, Cat# MF051-01). Real-time PCR was performed in triplicate in 20 μL reaction mixtures, and real-time qPCR analysis was performed using ABI QuantStudio 7 Flex with SYBR kit (TransGen, Cat# AQ601-02). Each sample was tested in three biological replicates per assay. Relative gene expression levels were analyzed using the comparative threshold cycle (Ct) method, where Ct is the threshold number of cycles normalized to GAPDH, reflecting the mRNA level.

Primer sequence:

MDH2:
AAAGTAGCTGTGCTAGGGGC (Former Primer 5′-3′),
GGTCCGAGGTAGCCTTTCAC (Reverse Primer 5′-3′).
GPX4:
GAGGCAAGACCGAAGTAAACTAC (Former Primer 5′-3′),
CCGAACTGGTTACACGGGAA (Reverse Primer 5′-3′).
SLC7A11:
GGTCCATTACCAGCTTTTGTACG (Former Primer 5′-3′),
AATGTAGCGTCCAAATGCCAG (Reverse Primer 5′-3′).
GAPDH:
GAAGGTCGGAGTCAACGGAT (Former Primer 5′-3′),
GACGGTGCCATGGAATTTGC (Reverse Primer 5′-3′).

### 4.9. Co-Immunoprecipitation (CO-IP) and Immunoblotting Analysis

Cells were harvested and lysed with RIPA cell lysis buffer (Solarbio, Cat# R0100) containing 1% protease inhibitor PMSF (Dingguo Biotechnology, Cat# 329-98-6) for 30 min at 4 °C, and then cell lysates were transferred into a 1.5 mL EP tube (Eppendorf tube) and centrifuged at 12,500× *g* for 20 min at 4 °C. After centrifugation, the supernatant was transferred to a new EP tube, mixed with 50 μL of protein G-agarose beads and 1 μg of antibody, and incubated for 4 h at 4 °C. After incubation, samples were centrifuged at 780× *g* for 2 min, the supernatant was discarded, and the immunoprecipitate was washed three times with RIPA cell lysis buffer (Solarbio, Cat# R0100). Finally, the immunoprecipitate was dissolved in SDS buffer and boiled for 10 min before performing SDS-PAGE.

### 4.10. Ubiquitination Assay

HEK-293T cells were seeded and transiently transfected with plasmids (HA-Ub, Myc-MDH2, and Flag-GPX4). Forty-eight hours after transfection, cells were treated with 10 μM MG132 (Biovision, Cat# 1791-5) for 6 h, and then cells were harvested and lysed with RIPA cell lysis buffer (radio-immunoprecipitation assay buffer, Solarbio, Cat# R0100) containing 1% PMSF (Dingguo Biotechnology, Cat# 329-98-6), 1% SDS (Solarbio, Cat# S8010), and 1% NEM (Solarbio, Cat# N8760), and immunoprecipitation was performed with anti-Flag antibodies(Sigma-Aldrich, Cat# 66008-3Ig). Immunoprecipitated proteins were analyzed by WB using anti-HA antibodies (Santa Cruz, Cat# sc-7392).

### 4.11. Cell Death Analysis

Cell death analysis was determined by PI staining. Briefly, cells were harvested, rinsed with PBS, and subsequently incubated with 10 μg/mL PI (TargetMol, Cat# T2130) for 15 min, at room temperature, in the absence of light. After staining, cells were centrifuged and washed twice with PBS to eliminate excess PI. The stained cells were then resuspended in 500 μL PBS, chilled on ice, and subjected to flow cytometry analysis. Data analysis was performed using FlowJo 10 software.

### 4.12. Cell Viability Assay

Cell viability was determined by CCK-8 assay. Briefly, cells were digested and counted, and 3500 cells in 200 μL DMEM (Solarbio, Cat# 11965) were seeded into 96-well plates and cultured under different conditions. After the culture, 10 μL of CCK-8 reagent (TargetMol, Cat# C0005) was added to each well, and the plates were incubated in the incubator for 1 h. The OD value of each sample was measured at 450 nm on a microplate reader, and the cell viability rate was calculated.

### 4.13. Intracellular Reactive Oxygen Species (ROS), Fe^2+^, and Lipid ROS Levels Detection

Intracellular ROS, Fe^2+^, and lipid ROS levels were determined using flow cytometry, according to the manufacturer’s instructions. Briefly, cells were seeded and cultured in 6-well plates, the culture medium was removed, and the cells were washed three times with PBS. Then, cells were treated with ROS probe DCFH-DA (MCE, Cat# HY-D0940), Fe^2+^ probe PGSK (MCE, Cat# HY-126823), and lipid ROS probe Cll-BODIPY^581/591^ (MCE, Cat# HY-D1691) in PBS and cultured in the cell incubator for 30 min. After incubation, cells were digested, collected, and washed twice with PBS and finally resuspended in 500 μL PBS on ice and analyzed with flow cytometry. Data were analyzed by FlowJo 10 software.

### 4.14. Statistical Analysis

GraphPad Prism 9 was used for all statistical analyses. The quantitative data presented represent the mean ±  standard deviation (SD) of a minimum of three independent experiments. Two-group comparisons were performed using the unpaired Student’s *t*-test. For comparisons among multiple groups, one-way ANOVA was used to compare the difference. Two-way ANOVA was used to calculate differences between two curves with multiple time or concentration points. Pearson correlation analysis was used to calculate the correlation coefficient. A *p*-value (alpha-value) of less than 0.05 was considered to indicate statistical significance (* *p* < 0.05, ** *p* < 0.01, and *** *p* < 0.001). A *p*-value > 0.05 was considered not significant and was denoted by “ns”.

## 5. Conclusions

Our study provides new insights into the role of MDH2 in HCC, demonstrating its function in inhibiting ferroptosis through GPX4 stabilization, thereby promoting HCC cell growth. These findings offer novel targets and strategies for HCC treatment. Further investigations into the regulatory mechanisms of MDH2 in HCC development and exploration of therapeutic approaches targeting MDH2 and ferroptosis are warranted.

## Data Availability

The data presented in this study are available upon request from the corresponding author.

## References

[1] Wu Z., Xia F., Wang W., Zhang K., Fan M., Lin R. (2024). Worldwide burden of liver cancer across childhood and adolescence, 2000–2021: A systematic analysis of the Global Burden of Disease Study 2021. eClinicalMedicine.

[2] Abboud Y., Shah V.P., Bebawy M., Al-Khazraji A., Hajifathalian K., Gaglio P.J. (2024). Mapping the Hidden Terrain of Hepatocellular Carcinoma: Exploring Regional Differences in Incidence and Mortality across Two Decades by Using the Largest US Datasets. J. Clin. Med..

[3] Lai Y.-W., Chung C.-H. (2024). Epidemiology of Hepatocellular Carcinoma in Taiwan. Clin. Pract..

[4] Lurje I., Czigány Z., Bednarsch J., Roderburg C., Isfort P., Neumann U., Lurje G. (2019). Treatment Strategies for Hepatocellular Carcinoma-A Multidisciplinary Approach. Int. J. Mol. Sci..

[5] Wang M.-D., Diao Y.-K., Yao L.-Q., Fan Z.-Q., Wang K.-C., Wu H., Gu L.-H., Xu J.-H., Li C., Lv G.-Y. (2024). Emerging role of molecular diagnosis and personalized therapy for hepatocellular carcinoma. iLIVER.

[6] Dixon S.J., Lemberg K.M., Lamprecht M.R., Skouta R., Zaitsev E.M., Gleason C.E., Patel D.N., Bauer A.J., Cantley A.M., Yang W.S. (2012). Ferroptosis: An iron-dependent form of nonapoptotic cell death. Cell.

[7] Hadian K., Stockwell B.R. (2020). SnapShot: Ferroptosis. Cell.

[8] Xue Q., Yan D., Chen X., Li X., Kang R., Klionsky D.J., Kroemer G., Chen X., Tang D., Liu J. (2023). Copper-dependent autophagic degradation of GPX4 drives ferroptosis. Autophagy.

[9] Co H.K.C., Wu C.C., Lee Y.C., Chen S.H. (2024). Emergence of large-scale cell death through ferroptotic trigger waves. Nature.

[10] Zhang S., Xin W., Anderson G.J., Li R., Gao L., Chen S., Zhao J., Liu S. (2022). Double-edge sword roles of iron in driving energy production versus instigating ferroptosis. Cell Death Dis..

[11] Park M.W., Cha H.W., Kim J., Kim J.H., Yang H., Yoon S., Boonpraman N., Yi S.S., Yoo I.D., Moon J.S. (2021). NOX4 promotes ferroptosis of astrocytes by oxidative stress-induced lipid peroxidation via the impairment of mitochondrial metabolism in Alzheimer’s diseases. Redox Biol..

[12] De Domenico I., Ward D.M., di Patti M.C., Jeong S.Y., David S., Musci G., Kaplan J. (2007). Ferroxidase activity is required for the stability of cell surface ferroportin in cells expressing GPI-ceruloplasmin. Embo J..

[13] Sarkar J., Seshadri V., Tripoulas N.A., Ketterer M.E., Fox P.L. (2003). Role of ceruloplasmin in macrophage iron efflux during hypoxia. J. Biol. Chem..

[14] Yeh K.Y., Yeh M., Glass J. (2011). Interactions between ferroportin and hephaestin in rat enterocytes are reduced after iron ingestion. Gastroenterology.

[15] Wang C., Xie L., Xing Y., Liu M., Yang J., Gao N., Cai Y. (2023). Iron-overload-induced ferroptosis in mouse cerebral toxoplasmosis promotes brain injury and could be inhibited by Deferiprone. PLoS Negl. Trop. Dis..

[16] Yang W.S., SriRamaratnam R., Welsch M.E., Shimada K., Skouta R., Viswanathan V.S., Cheah J.H., Clemons P.A., Shamji A.F., Clish C.B. (2014). Regulation of ferroptotic cancer cell death by GPX4. Cell.

[17] Ingold I., Berndt C., Schmitt S., Doll S., Poschmann G., Buday K., Roveri A., Peng X., Porto Freitas F., Seibt T. (2018). Selenium Utilization by GPX4 Is Required to Prevent Hydroperoxide-Induced Ferroptosis. Cell.

[18] Shah R., Shchepinov M.S., Pratt D.A. (2018). Resolving the Role of Lipoxygenases in the Initiation and Execution of Ferroptosis. ACS Cent. Sci..

[19] Wang L., Liu Y., Du T., Yang H., Lei L., Guo M., Ding H.F., Zhang J., Wang H., Chen X. (2020). ATF3 promotes erastin-induced ferroptosis by suppressing system Xc^−^. Cell Death Differ..

[20] Sui X., Zhang R., Liu S., Duan T., Zhai L., Zhang M., Han X., Xiang Y., Huang X., Lin H. (2018). RSL3 Drives Ferroptosis Through GPX4 Inactivation and ROS Production in Colorectal Cancer. Front. Pharmacol..

[21] Li D., Li Y., Chen L., Gao C., Dai B., Yu W., Yang H., Pi J., Bian X. (2024). Natural Product Auraptene Targets SLC7A11 for Degradation and Induces Hepatocellular Carcinoma Ferroptosis. Antioxidants.

[22] Chen Y., Li L., Lan J., Cui Y., Rao X., Zhao J., Xing T., Ju G., Song G., Lou J. (2022). CRISPR screens uncover protective effect of PSTK as a regulator of chemotherapy-induced ferroptosis in hepatocellular carcinoma. Mol. Cancer.

[23] Dupourque D., Kun E. (1969). Malate dehydrogenases of ox kidney. 2. Two substrate kinetic and inhibition analyses. Eur. J. Biochem..

[24] Cannon-Albright L.A., Stevens J., Teerlink C.C., Facelli J.C., Allen-Brady K., Welm A.L. (2023). A Rare Variant in MDH2 (rs111879470) Is Associated with Predisposition to Recurrent Breast Cancer in an Extended High-Risk Pedigree. Cancers.

[25] Zhuang Y., Xiang J., Bao W., Sun Y., Wang L., Tan M., He Y., Xi X. (2017). MDH2 Stimulated by Estrogen-GPR30 Pathway Down-Regulated PTEN Expression Promoting the Proliferation and Invasion of Cells in Endometrial Cancer. Transl. Oncol..

[26] Lo Y.W., Lin S.T., Chang S.J., Chan C.H., Lyu K.W., Chang J.F., May E.W., Lin D.Y., Chou H.C., Chan H.L. (2015). Mitochondrial proteomics with siRNA knockdown to reveal ACAT1 and MDH2 in the development of doxorubicin-resistant uterine cancer. J. Cell Mol. Med..

[27] Zhang B., Tornmalm J., Widengren J., Vakifahmetoglu-Norberg H., Norberg E. (2017). Characterization of the Role of the Malate Dehydrogenases to Lung Tumor Cell Survival. J. Cancer.

[28] Ma Y.C., Tian P.F., Chen Z.P., Yue D.S., Liu C.C., Li C.G., Chen C., Zhang H., Liu H.L., Zhang Z.F. (2021). Urinary malate dehydrogenase 2 is a new biomarker for early detection of non-small-cell lung cancer. Cancer Sci..

[29] Liu Q., Harvey C.T., Geng H., Xue C., Chen V., Beer T.M., Qian D.Z. (2013). Malate dehydrogenase 2 confers docetaxel resistance via regulations of JNK signaling and oxidative metabolism. Prostate.

[30] Vujicic I., Rusevski A., Stankov O., Popov Z., Dimovski A., Davalieva K. (2022). Potential Role of Seven Proteomics Tissue Biomarkers for Diagnosis and Prognosis of Prostate Cancer in Urine. Diagnostics.

[31] Gao J., Zhou Q., Wu D., Chen L. (2021). Mitochondrial iron metabolism and its role in diseases. Clin. Chim. Acta.

[32] Nie G., Sheftel A.D., Kim S.F., Ponka P. (2005). Overexpression of mitochondrial ferritin causes cytosolic iron depletion and changes cellular iron homeostasis. Blood.

[33] Sandoval-Acuña C., Torrealba N., Tomkova V., Jadhav S.B., Blazkova K., Merta L., Lettlova S., Adamcová M.K., Rosel D., Brábek J. (2021). Targeting Mitochondrial Iron Metabolism Suppresses Tumor Growth and Metastasis by Inducing Mitochondrial Dysfunction and Mitophagy. Cancer Res..

[34] Chen Y., Zhang Y., Zhang S., Ren H. (2024). Molecular insights into sarcopenia: Ferroptosis-related genes as diagnostic and therapeutic targets. J. Biomol. Struct. Dyn..

[35] Hou P.P., Luo L.J., Chen H.Z., Chen Q.T., Bian X.L., Wu S.F., Zhou J.X., Zhao W.X., Liu J.M., Wang X.M. (2020). Ectosomal PKM2 Promotes HCC by Inducing Macrophage Differentiation and Remodeling the Tumor Microenvironment. Mol. Cell.

[36] Bian X.L., Chen H.Z., Yang P.B., Li Y.P., Zhang F.N., Zhang J.Y., Wang W.J., Zhao W.X., Zhang S., Chen Q.T. (2017). Nur77 suppresses hepatocellular carcinoma via switching glucose metabolism toward gluconeogenesis through attenuating phosphoenolpyruvate carboxykinase sumoylation. Nat. Commun..

[37] Xu D., Shao F., Bian X., Meng Y., Liang T., Lu Z. (2021). The Evolving Landscape of Noncanonical Functions of Metabolic Enzymes in Cancer and Other Pathologies. Cell Metab..

[38] Jiang X., Stockwell B.R., Conrad M. (2021). Ferroptosis: Mechanisms, biology and role in disease. Nat. Rev. Mol. Cell Biol..

[39] Hassannia B., Vandenabeele P., Vanden Berghe T. (2019). Targeting Ferroptosis to Iron Out Cancer. Cancer Cell.

[40] Badgley M.A., Kremer D.M., Maurer H.C., DelGiorno K.E., Lee H.J., Purohit V., Sagalovskiy I.R., Ma A., Kapilian J., Firl C.E.M. (2020). Cysteine depletion induces pancreatic tumor ferroptosis in mice. Science.

[41] Koppula P., Zhang Y., Zhuang L., Gan B. (2018). Amino acid transporter SLC7A11/xCT at the crossroads of regulating redox homeostasis and nutrient dependency of cancer. Cancer Commun..

[42] Liu Q., Ding X., Xu X., Lai H., Zeng Z., Shan T., Zhang T., Chen M., Huang Y., Huang Z. (2022). Tumor-targeted hyaluronic acid-based oxidative stress nanoamplifier with ROS generation and GSH depletion for antitumor therapy. Int. J. Biol. Macromol..

[43] Tang D., Kroemer G. (2020). Ferroptosis. Curr. Biol..

[44] Xu C., Sun S., Johnson T., Qi R., Zhang S., Zhang J., Yang K. (2021). The glutathione peroxidase Gpx4 prevents lipid peroxidation and ferroptosis to sustain Treg cell activation and suppression of antitumor immunity. Cell Rep..

[45] Ozkan E., Bakar-Ates F. (2022). Ferroptosis: A Trusted Ally in Combating Drug Resistance in Cancer. Curr. Med. Chem..

[46] Mao C., Liu X., Zhang Y., Lei G., Yan Y., Lee H., Koppula P., Wu S., Zhuang L., Fang B. (2021). DHODH-mediated ferroptosis defence is a targetable vulnerability in cancer. Nature.

[47] Bi G., Liang J., Bian Y., Shan G., Huang Y., Lu T., Zhang H., Jin X., Chen Z., Zhao M. (2024). Polyamine-mediated ferroptosis amplification acts as a targetable vulnerability in cancer. Nat. Commun..

[48] Hangauer M.J., Viswanathan V.S., Ryan M.J., Bole D., Eaton J.K., Matov A., Galeas J., Dhruv H.D., Berens M.E., Schreiber S.L. (2017). Drug-tolerant persister cancer cells are vulnerable to GPX4 inhibition. Nature.

[49] Reisch A.S., Elpeleg O. (2007). Biochemical assays for mitochondrial activity: Assays of TCA cycle enzymes and PDHc. Methods Cell Biol..

[50] Jiang X., Peng Q., Peng M., Oyang L., Wang H., Liu Q., Xu X., Wu N., Tan S., Yang W. (2024). Cellular metabolism: A key player in cancer ferroptosis. Cancer Commun..

[51] Zhao L., Zhao J., Zhong K., Tong A., Jia D. (2022). Targeted protein degradation: Mechanisms, strategies and application. Signal Transduct. Target. Ther..

[52] Yan H.F., Zou T., Tuo Q.Z., Xu S., Li H., Belaidi A.A., Lei P. (2021). Ferroptosis: Mechanisms and links with diseases. Signal Transduct. Target. Ther..

[53] Díaz-Castro J., Pulido M., Alférez M.J., Ochoa J.J., Rivas E., Hijano S., López-Aliaga I. (2014). Goat milk consumption modulates liver divalent metal transporter 1 (DMT1) expression and serum hepcidin during Fe repletion in Fe-deficiency anemia. J. Dairy Sci..

[54] Howitt J., Putz U., Lackovic J., Doan A., Dorstyn L., Cheng H., Yang B., Chan-Ling T., Silke J., Kumar S. (2009). Divalent metal transporter 1 (DMT1) regulation by Ndfip1 prevents metal toxicity in human neurons. Proc. Natl. Acad. Sci. USA.

[55] Wolff N.A., Garrick M.D., Zhao L., Garrick L.M., Ghio A.J., Thévenod F. (2018). A role for divalent metal transporter (DMT1) in mitochondrial uptake of iron and manganese. Sci. Rep..

[56] Lee J.S., Kang J.H., Lee S.H., Hong D., Son J., Hong K.M., Song J., Kim S.Y. (2016). Dual targeting of glutaminase 1 and thymidylate synthase elicits death synergistically in NSCLC. Cell Death Dis..

[57] Mukhopadhyay S., Goswami D., Adiseshaiah P.P., Burgan W., Yi M., Guerin T.M., Kozlov S.V., Nissley D.V., McCormick F. (2020). Undermining Glutaminolysis Bolsters Chemotherapy While NRF2 Promotes Chemoresistance in KRAS-Driven Pancreatic Cancers. Cancer Res..

